# Improving Large Area Population Mapping Using Geotweet Densities

**DOI:** 10.1111/tgis.12214

**Published:** 2016-06-30

**Authors:** Nirav N. Patel, Forrest R. Stevens, Zhuojie Huang, Andrea E. Gaughan, Iqbal Elyazar, Andrew J. Tatem

**Affiliations:** ^1^ Department of Geography and Geoinformation ScienceGeorge Mason UniversityFairfax; ^2^ Department of Geography and GeosciencesUniversity of Louisville; ^3^ Department of GeographyGeoVISTA Center and Centre for Infectious Disease Dynamics, Pennsylvania State University; ^4^ Eijkman‐Oxford Clinical Research UnitJakarta; ^5^ WorldPop Project, Department of Geography and EnvironmentUniversity of Southampton; ^6^ Fogarty International CenterNational Institutes of Health; ^7^ Flowminder FoundationStockholm

## Abstract

Many different methods are used to disaggregate census data and predict population densities to construct finer scale, gridded population data sets. These methods often involve a range of high resolution geospatial covariate datasets on aspects such as urban areas, infrastructure, land cover and topography; such covariates, however, are not directly indicative of the presence of people. Here we tested the potential of geo‐located tweets from the social media application, Twitter, as a covariate in the production of population maps. The density of geo‐located tweets in 1x1 km grid cells over a 2‐month period across Indonesia, a country with one of the highest Twitter usage rates in the world, was input as a covariate into a previously published random forests‐based census disaggregation method. Comparison of internal measures of accuracy and external assessments between models built with and without the geotweets showed that increases in population mapping accuracy could be obtained using the geotweet densities as a covariate layer. The work highlights the potential for such social media‐derived data in improving our understanding of population distributions and offers promise for more dynamic mapping with such data being continually produced and freely available.

## 1 Introduction

The global population is projected to increase from 7 billion to over 9 billion over the next four decades, with much of this growth concentrated in low‐income countries (UN 2011). The effects of such rapid demographic growth are well documented, with impacts on the economies, environment and health of nations (Bongaarts 2009). To measure the impact of this population growth, as well as progress towards development goals, there is a need for contemporary, spatially explicit, high resolution maps that accurately identify population distributions.

High‐income countries often have mapping expertise and substantial resources at their disposal to create accurate and contemporary spatial population datasets. However, across the lower income regions of the world, equivalent resources and relevant data can often be either lacking or are of poor quality (Tatem and Linard 2011). Over the past few decades there has been increasing interest in creating large‐area gridded population distribution datasets (Cheriyadat et al. 2007; Balk et al. 2006; Linard et al. 2012) to support applications such as disease burden estimation, climate change and human health adaptive strategies, disaster response, accessibility modelling, transport and city planning, and environmental impact assessment (Balk et al. 2006; Linard et al. 2010, 2012; McMichael et al. 2006; Rasul and Thapa 2003; Tatem, et al. 2007). Current global gridded population datasets include the Gridded Population of the World (GPW) database (Balk and Yetman 2004; Tobler et al. 1997) and the Global Rural Urban Mapping Project (GRUMP) (Balk et al. 2005). In addition, there is the LandScan Global Population database (Bhaduri et al. 2007; Dobson et al. 2000), and the United Nations Environment Programme (UNEP) compiled gridded datasets for Latin America, Africa, and Asia (Nelson 2004; Deichmann 1996). The WorldPop project(http://www.worldpop.org.uk/) provides freely available gridded population data for Africa, Asia and the Americas (Linard and Tatem 2012; Linard et al. 2010; Stevens et al. 2015). With the exception of GPW, all of these datasets use spatial covariate datasets on factors related to the way that humans distribute themselves on the landscape to disaggregate areal‐unit based census counts to grid squares.

Spatial covariate datasets used in the population disaggregation process tend to include factors known to correlate with population densities, such as satellite‐derived maps of human settlements, urban areas, topography, lights at night, and land cover. Additionally, infrastructure‐related variables have been used, including road networks and health facilities (e.g. Stevens et al. 2015). However, all of these covariates are typically static in nature and not direct measures of the presence of people. Recent efforts have shown the potential of “big data” sources, such as mobile phone call data records, to map populations dynamically using the communication patterns of phone users (Deville et al. 2014), but such data are generally difficult to obtain and are highly sensitive, both commercially and for privacy reasons. The rise in data availability of user communications and check‐ins through social media presents opportunities however, in terms of a data‐source that is freely available, dynamic and without the data sensitivity restrictions of mobile call data records. However it is important to consider that a limitation of utilizing social media is that Internet connected smartphones are often expensive resources in low‐income countries and the applicability of these methodologies might be limited (Ramaswamy et al. 2009).

One of the most popular social media applications over the past decade has been Twitter (https://twitter.com). Twitter is an online social networking service that allows users to post 140‐character messages called “tweets” to a publicly viewable microblog platform, and since its inception in 2006, the service has gained worldwide popularity. Despite the relative lack of tweets with geographic metadata (around 2.02% of tweets are posted with such metadata globally), many useful geographic applications have been derived from tweet data (Takhteyev et al. 2012; Leetaru et al. 2013; Hawelka et al. 2014; Blanford et al. 2015). The maps of geo‐located tweets in countries where Twitter is popular show detailed depictions of human activity, with the location of tweets indicative of settlements, transportation networks, and building locations (Leetaru et al. 2013). Such data therefore have the potential to provide a valuable ancillary covariate layer in the population mapping process, and also one that changes dynamically, but its utility has yet to be tested.

Here we assess the potential of geo‐located tweets to improve population distribution maps. Tweets are integrated as a covariate layer into a census data disaggregation model. We compare the accuracy of gridded population maps produced with and without geotweet data and discuss the advantages and disadvantages of such social media in improving population mapping accuracies in low and middle income settings.

## 2 Methods

### 2.1 Study Area

**Figure 1:**
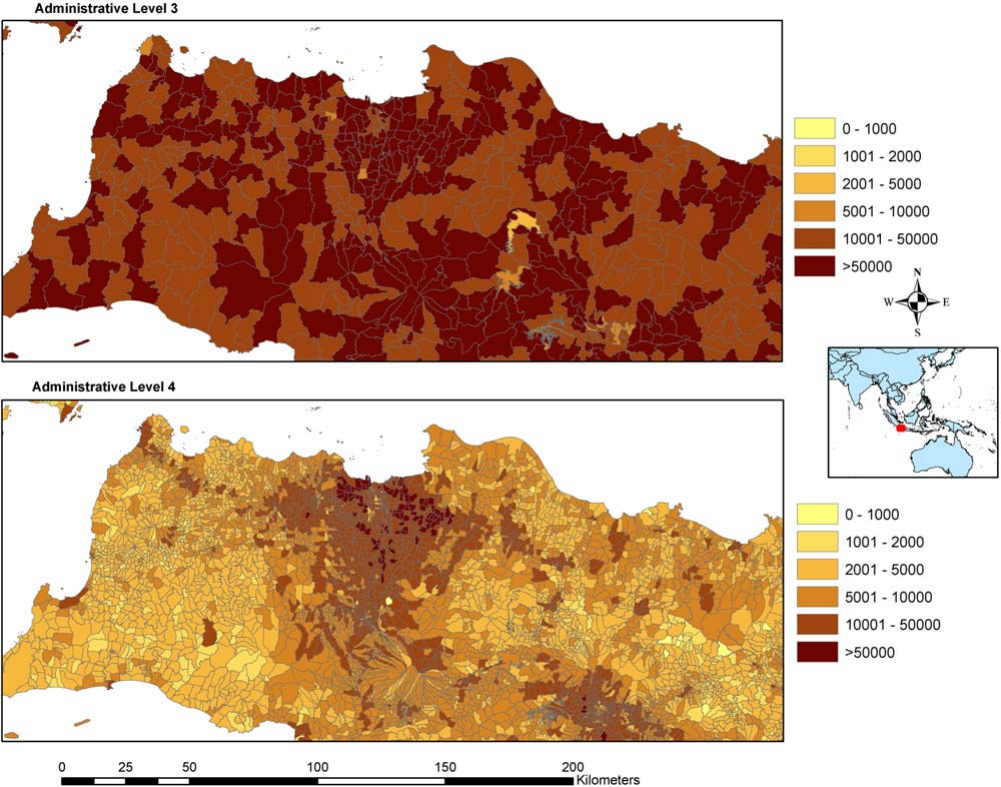
Map of Indonesia administrative boundaries levels 3 and 4, focused around Jakarta, with administrative units shaded to show population counts per administrative unit

Indonesia has one of the highest Twitter user levels in the world (Leetaru et al. 2013) and it also has recent, very high spatial resolution census data. These characteristics combined make the country an ideal case study for the utility of geotweet data for census count disaggregation. The study area is the country of Indonesia, an archipelago made up of thousands of islands, with a total land area of approximately 1.9 million km^2^. For the purposes of this study, boundary‐matched census data at the Kecamatan administrative level (Level 3, 6,463 units) and Desa/Kelurahan administrative level (Level 4, 79,618 units), were obtained (Figure 1).

### 2.2 Mapping Geotweets

Two months of geo‐located tweets (7/8–8/8 and 10/12–11/15, 2013) were extracted from the Twitter Streaming API (https://dev.twitter.com/streaming/overview) for Indonesia. Morstatter et al. (2013) show the Twitter Streaming API provides around 90.1% coverage of the total available set of geotagged tweets. Note that the geotagged tweets with exact latitude and longitude make up about 1.6% of the total number of tweets, which is about 79% of tweets posted with general geographic metadata; see Twitter Places attributes (https://dev.twitter.com/overview/api/places#attributes) (Leetaru et al. 2013). Similar to the process described in Morstatter et al. (2013), the data streaming was performed by utilizing the Tweetpy library (https://github.com/tweepy/tweepy) on an Amazon Web Service (http://aws.amazon.com/) Instance. Further reference on Twitter Streaming API and the most up‐to‐date usage agreements can be found at https://dev.twitter.com/.

**Figure 2:**
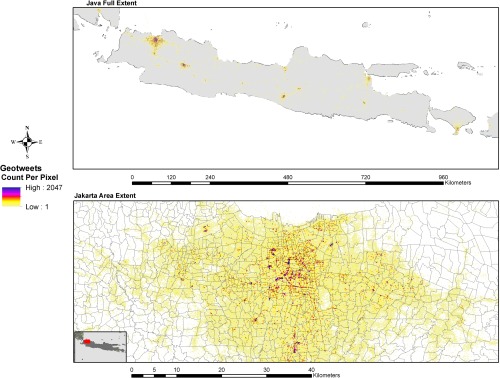
Results of a two‐month aggregation of geo‐located tweets over the full extent of Java (top) and a view focused on Jakarta (bottom)

The collected data was automatically uploaded to a storage bucket on Amazon Simple Storage Services (S3) (http://aws.amazon.com/s3/) every day during the study period. An Apache Pig (https://pig.apache.org/) process was initiated on the Amazon Elastic MapReduce (http://aws.amazon.com/elasticmapreduce/) web service to extract all the tweets within the geographic boundary constraint of Indonesia and to aggregate the raw tweet activity counts into a 0.001 by 0.001 degree grid in an unprojected geographic coordinate system. During the aggregation process, the origin latitude and longitude from the geotagged tweets were rounded down to three decimal digits. The list with latitude, longitude and tweet counts were imported into ArcGIS Desktop (http://www.esri.com/software/arcgis/arcgis-for-desktop) to produce a raster layer for testing as a covariate in population mapping (Figure 2).

The geotweets raster dataset was then integrated into a population mapping process along with other ancillary covariates to disaggregate the administrative unit level 3 census data to a 100 by 100 m grid using a population mapping process detailed in the next section.

### 2.3 High Resolution Population Mapping Method

The population mapping method detailed in Stevens et al. (2015) was utilized to undertake two tests – mapping the entire Republic of Indonesia, and disaggregating administrative unit level 3 data with and without the extracted geotweets to assess whether the inclusion of the tweets improved mapping accuracies when compared with the administrative level 4 data.

#### 2.3.1 Data processing

Indonesian census counts for 2010 were obtained from the Indonesian Government and matched to GIS‐administrative boundaries at administrative level 3 (6,463 units, total spatial area calculation from shapefile = 3,364,560.063 km^2^) and administrative level 4 (79,618 units, total spatial area calculation from shapefile = 3,362,579.043 km^2^). Both data sets have a total population of 243,530,782 and an average spatial resolution (ASR) of 22.8 and 6.50, for the administrative level 3 and the administrative level 4, respectively. The ASR is calculated as the square root of its surface area (in square kilometers) divided by the number of total administrative units (Balk and Yetman 2004) and provides a broad measure of mean administrative unit size across the country. When calculated by province (administrative unit level 2), the ASR varies from 1.68 to 68.9 for level 3 units and from 0.919 to 30.8 for level 4 units. The administrative level 3 census data was used in the actual model implementation while the administrative level 4 data was held in reserve for model assessment purposes.

The modeling process uses a suite of continuous and discrete data layers to generate an estimated population density‐weighting layer. The majority of these data sets are contemporary and freely available (Table 1). The rationale behind using the datasets detailed in Table 1 is to include geospatial data that may correlate with human population presence on the landscape as cited in Stevens et al. (2015).

**Table 1:** Test‐specific data sources and variable names used for population density estimation with dasymetric weights

Type	Variable Name(s)*	Description	Indonesia Data
Census		Country‐specific census data that isused for disaggregation	2010, Admin‐level 3 and Admin‐level 4 (census datasets received from the Government of Indonesia)
Land Cover	lan_cls011, lan_dst011	Cultivated terrestrial lands	Landcover utilizing 3‐year Google Earth Engine data & MDA GlobCover with methods from Patel et al. (2015).
	lan_cls040, lan_dst040	Woody/Trees	
	lan_cls130, lan_dst130	Shrubs	
	lan_cls140, lan_dst140	Herbaceous	
	lan_cls150, lan_dst150	Other terrestrial vegetation	
	lan_cls160, lan_dst160	Aquatic vegetation	
	lan_cls190, lan_dst190	Urban area	
	lan_cls200, lan_dst200	Bare areas	
	lan_cls210, lan_dst210	Water bodies	
	lan_cls230, lan_dst230	No data, cloud/shadow	
	lan_cls240, lan_dst240	Rural settlement	
	lan_cls250, lan_dst250	Industrial area	
	lan_clsBLT, lan_dstBLT	Built, merged urban/rural class	
Continuous			
Raster‐Format			
	Lig	Lights at night data	Suomi VIIRS‐Derived (NOAA 2012)
	Npp	MODIS 17A3 2010 estimated net primary productivity, 1 km	Extraction from MODIS package in R (Running et al. 2004)
	Tem	Mean temperature, 1950–2000	WorldClim/BioClim (Hijmans et al. 2005)
	Pre	Mean precipitation, 1950–2000	WorldClim/BioClim (Hijmans et al. 2005)
	Ele	Elevation	HydroSHEDS (Lehner et al. 2006)
	ele_slope	Slope	HydroSHEDS‐Derived (Lehner et al. 2006)
	Twe	Tweets	Tweets data obtained from method detailed in Section 2.2
Converted			
Vector‐Format	roa_cls, roa_dst	Roads	OSM (2014)
	riv_dst	Distance to rivers/streams	VMAP0 merged†
	pop_cls, pop_dst	Populated Places	OSM (2014)
	wat_cls, wat_dst	Water bodies	VMAP0 merged†
	pro_cls, pro_dst	Protected areas	IUCN and UNEP (2012)
	poi_cls, poi_dst	Populated Points of Interest	OSM (2014)
	bui_cls, bui_dst	Buildings	OSM (2014)
	use_cls, use_dst	Delineated land uses	OSM (2014)
	cit_cls, cit_dst	Cities	OSM (2014)
	dwe_cls, dwe_dst	Dwellings	OSM (2014)
	ham_cls, ham_dst	Hamlets	OSM (2014)
	hos_cls, hos_dst	Hospital	OSM (2014)
	loc_cls, loc_dst	Localities	OSM (2014)
	pol_cls, pol_dst	Police	OSM (2014)
	sch_cls, sch_dst	Schools	OSM (2014)
	sub_cls, sub_dst	Suburbs	OSM (2014)
	tow_cls, tow_dst	Towns	OSM (2014)
	vil_cls, vil_dst	Villages	OSM (2014)
	ind_cls, ind_dst	Industrial land use	OSM (2014)
	res_cls, res_dst	Residential land use	OSM (2014)
	pri_cls, pri_dst	Primary roads	OSM (2014)
	sec_cls, sec_dst	Secondary roads	OSM (2014)
	ter_cls, ter_dst	Tertiary roads	OSM (2014)
	rro_cls, rro_dst	Residential roads	OSM (2014)
	ser_cls, ser_dst	Service roads	OSM (2014)
	nei_cls, nei_dst	Neighborhoods	OSM (2014)

*The variable names are used in the Random Forest model output and throughout the text to refer to the specific data they were derived from. The first three letters are derived from the data type (e.g. “lan” indicates land cover) and the last three letters, if present, indicates what type of data each variable represents (e.g. “_cls” is a binary classification and “_dst” is a calculated Euclidean distance‐to variable.

†The default data for populated places is merged from several VMAP0 data sources. There are three VMAP0 data sets used: The point data pop/builtupp and pop/mispopp are buffered to 100 m and merged with the pop/builtupa polygons creating a vector‐based built layer. This layer is then converted to binary class and distance‐to rasters for use in modeling (NGA 2005).

The MDA‐land cover data was modified with the inclusion of a classified urban/rural land cover informed by an urban extent delineation using the Google Earth Engine platform (Patel et al. 2015). The resulting infusion of the binary urban/rural raster layer represents an improved “built” class within the MDA land cover classes.

Each covariate layer contributes to a better understanding of landscape features across Indonesia, both natural and man‐made, as each may relate to population densities. In addition, to assess the added value of including spatially‐explicit social media data as a covariate in the model the best available ancillary data were combined with the geotweets. To compare model output and accuracy we create one set of outputs with geotweets included and one set of outputs without geotweets, both using the administrative level 3 census data.

In addition to census data, MDA‐derived land cover and additional covariates included those outlined in prior Random Forest‐based WorldPop datasets (Stevens et al. 2015). Raster‐based covariates included the HydroSheds‐based digital elevation data (also converted to slope estimates), the Suomi VIIRS‐derived lights at night raster layer, MODIS‐derived estimates of net primary productivity, WorldClim average temperature and precipitation data, and of course the custom geo‐located tweets. Vector‐based covariates, which are then processed to raster‐based derived products (Stevens et al. 2015) include Open Street Map derived datasets, NGA data (NGA Vector Map 2005) and the World Database of Protected Areas boundaries. These are outlined and cited in Table 1.

#### 2.3.2 Random forest population disaggregation method

**Figure 3:**
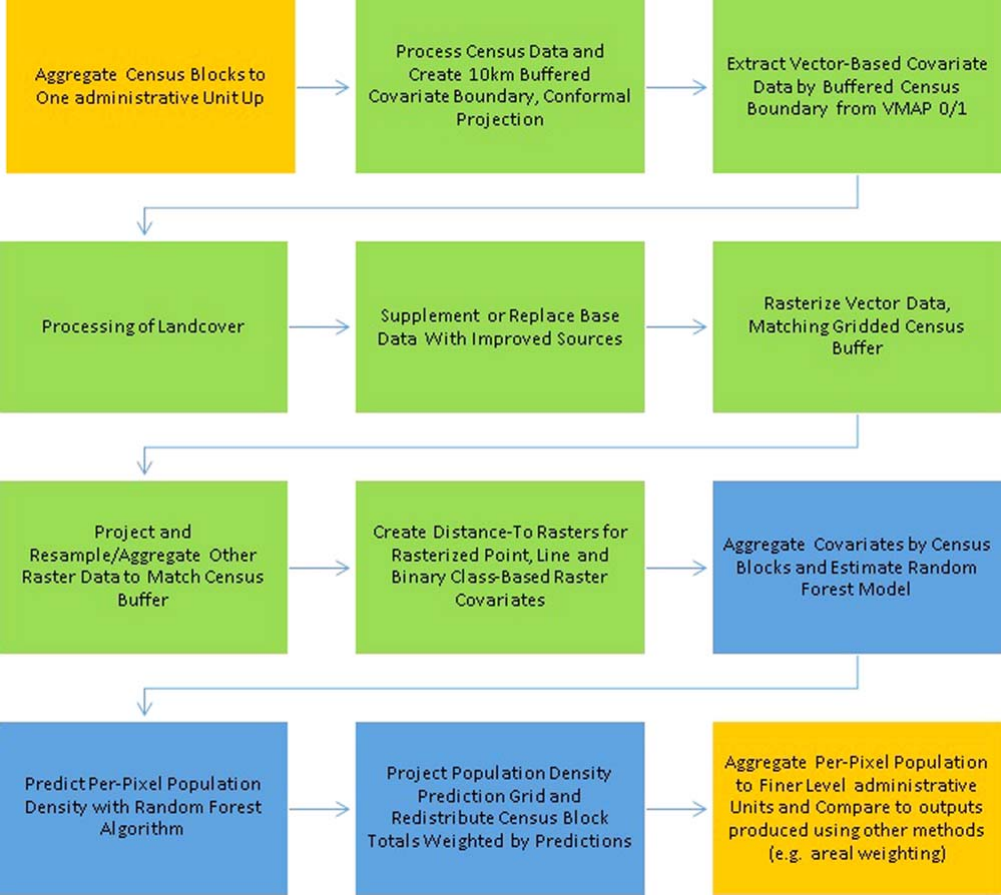
General structure of the data processing and map production procedure used to compare the methodology outlined in Stevens et al (2015). The orange boxes represent items that are specific to the research presented here and not part of end‐user map data product generation. The green boxes represent data pre‐processing stages. Items in blue represent Random Forest model estimation, per‐pixel prediction and dasymetric redistribution of census counts

The general process used for data preparation, modeling and validation for the population mapping is documented in Stevens et al. (2015). In brief, the aggregated population counts and the raster and vector layers shown in Table 1 are used to create a Random Forest‐based model (Breiman 2001), parameterized on census unit population densities to estimate population density using the ancillary covariates. The Random Forest (RF) algorithm, as a non‐parametric, ensemble statistical approach, provides flexibility in the modeling process for inclusion of disparate data types (Breiman 2001). The process involves growing a “forest” by generating individual, unpruned decision trees that are then aggregated to represent a final prediction for each grid cell in the weighting layer (Breiman 2001; Liaw and Wiener 2002). The resulting population density map is then used as a weighting layer for a standard dasymetric mapping approach as described for WorldPop population map products (Stevens et al. 2015; Gaughan et al. 2013, 2015; Linard et al. 2012; Linard and Tatem, 2012; Tatem et al. 2007). This process is depicted in Figure 3.

The RF model includes an internal cross‐validation component that provides additional insight into the prediction error of the model. During the estimation of the random forest, at each node of each tree, one‐third of the data is held in reserve from the iterative, bootstrapping process and used to generate an out‐of‐bag (OOB) error. The OOB error provides an unbiased estimate of prediction error for new, non‐reference data points (assuming those points contain covariate data that fall within combinations present in the training data). Another metric that is provided post‐hoc from the forest‐growing algorithm is the variable importance measures for each model which are presented as the mean decrease in the residual sum of squares OOB estimates when the variable is included in the tree split.

#### 2.3.3 Accuracy assessment

The population mapping was undertaken using administrative level three census data as input, then the output high resolution map was aggregated at administrative level four and compared with the counts at this level, following Gaughan et al. (2013) and Stevens et al. (2015). Summary statistics were calculated, including root mean square error (RMSE), the RMSE divided by the mean census unit count (%RMSE) and the mean absolute error (MAE). Together these statistics were used to compare the predictive ability of the mapping with and without the geotweets.

## 3 Results

### 3.1 Random Forest Statistical Outputs

**Figure 4:**
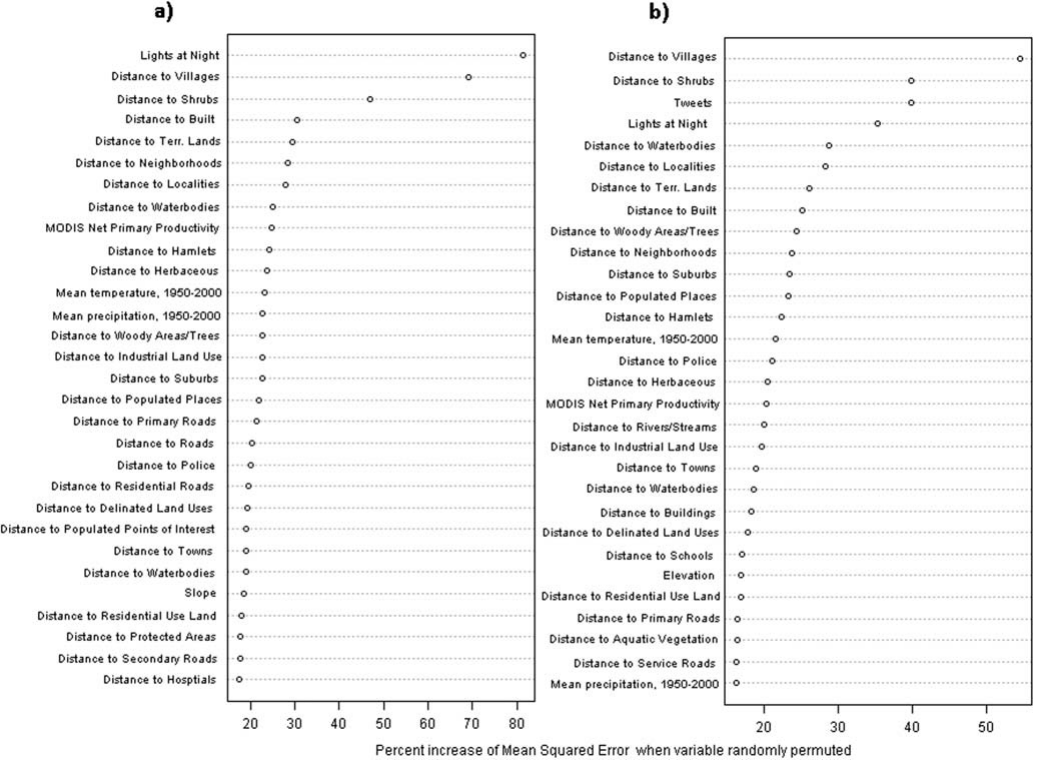
Covariate importance plots for tests: (a) without geotweets; and (b) with geotweets

Figure 4 shows the importance of the variables outlined in Table 1 in the mapping process as estimated by the increase in mean squared error (MSE) when the specified covariate is randomly permuted and predictions re‐calculated for OOB data. The most important variables include the built land cover covariates, indicating “Built” areas, which include urban and rural settlements that were created using the processes detailed in Section 2.3.1. The built covariates as well as the Suomi NPP Lights‐At‐Night‐derived covariate have been documented in previous literature as strong indicators of population (Patel et al. 2015). Here, the Lights‐At‐Night and Distance to Villages (derived from Open Street Map data) variables are the most important predictors in the model without geotweets. When the geotweets are included in the modelling process (Figure 4b), differing covariates become key contributors, and the geotweets density variable enters into the top three most important predictors. In comparing the performance of both models, the test without tweets could explain 93% of the variance within the RF model, and the test with tweets could explain 94% of the variance within its RF model.

**Figure 5:**
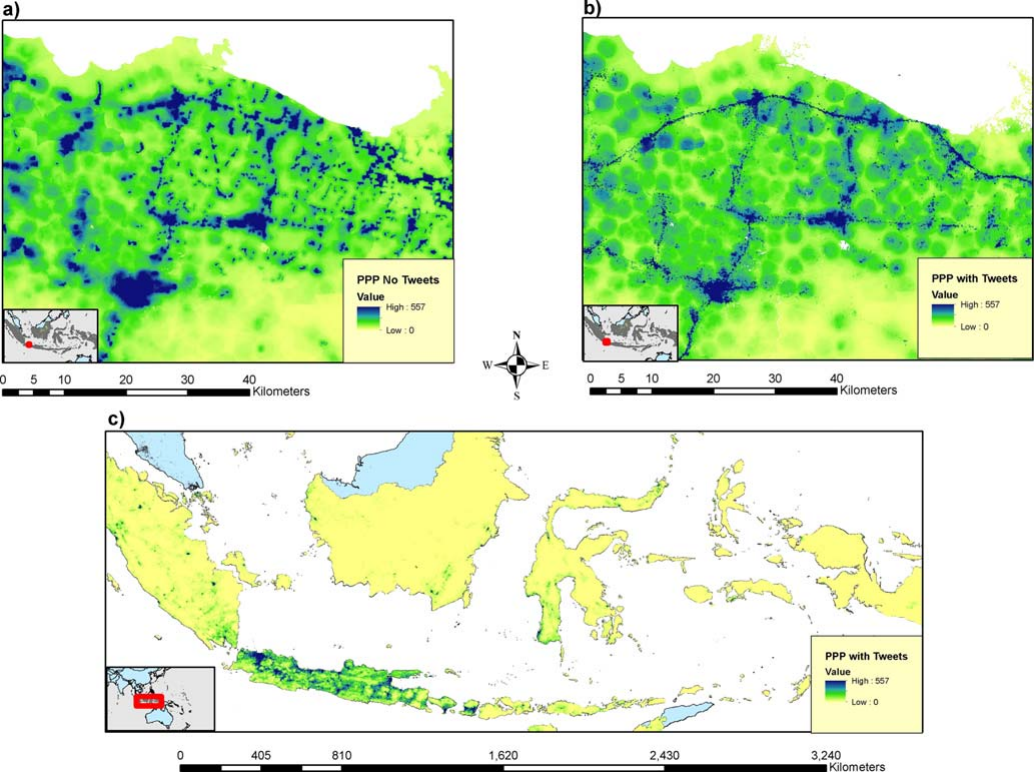
Map of Persons Per Pixel (PPP) produced using high resolution population mapping method (Stevens et al. 2015), showing the final population maps for a region on the island of Java with: (a) no geotweet data; (b) geotweet data included; and finally (c) the output dataset for the entirety of Indonesia with geotweet data included

Figure 5 shows visual examples of the mapping without (Figure 5a) and with (Figures 5b, c) the inclusion of the geotweet density covariate. Visually the maps are very different, with the geo‐located tweet inclusion resulting in a more constrained and higher density mapping of population density, specifically clustered around settlements and transportation networks.

### 3.2 External Accuracy Assessment

Table 2 presents the results of the comparison of administrative level 3 census data based mapping with and without geotweets against administrative level 4 census counts. The inclusion of the geotweet data as a covariate produced a significant reduction in estimation error for both the root mean square error (RMSE) and mean absolute error (MAE) when considering the sheer difference in this particular comparison on the amount of census units in administrative level 3 versus administrative level 4. The results in Table 2 indicate that the inclusion of geotweets reduced errors relative to the model without the geotweets data.

**Table 2:** Accuracy assessment results for tests

	RMSE (persons)	%RMSE	MAE (persons)
Admin 3 without tweets	2284.14	74.58	1123.44
Admin 3 with tweets	2213.99	72.29	1120.16
Difference (Without ‐ With)	70.15	2.29	3.28

**Figure 6:**
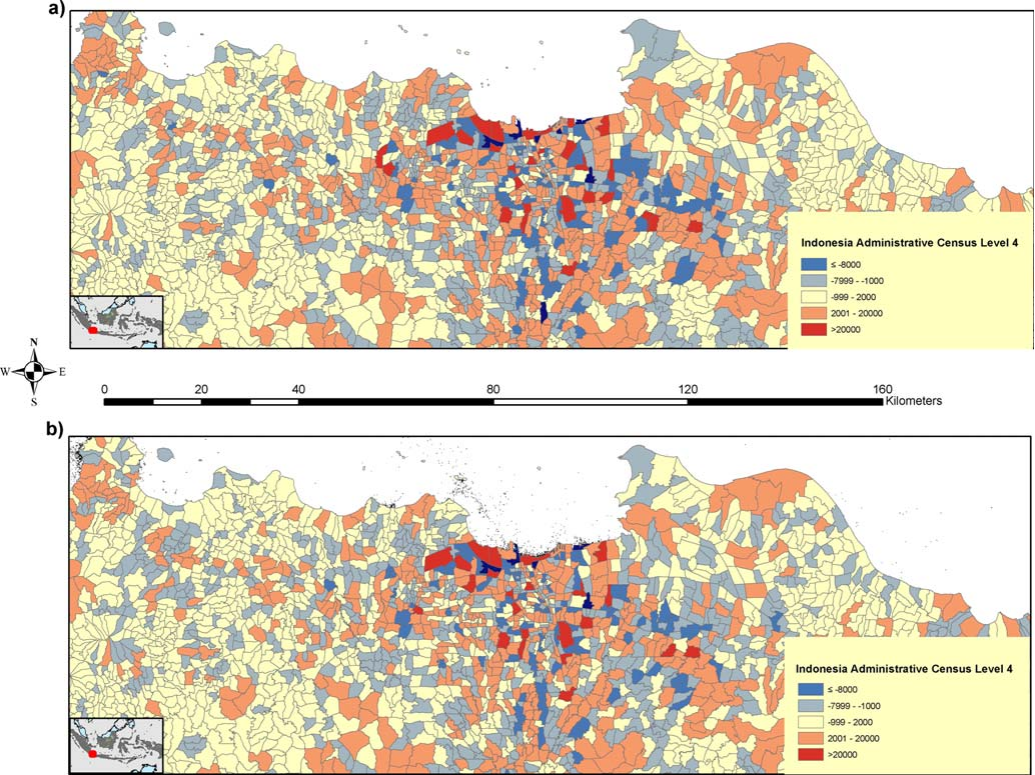
Differenced map produced through comparing the population maps generated with: (a) no geotweet data; and (b) geotweet data constructed from administrative level 3 census population counts and differencing the zonal sums against administrative level 4 census population count data

Figure 6 shows the results from comparing the geotweet and non‐geotweet population maps constructed using administrative unit level 3 population count data and applying zonal statistics to see how they compare with the finer administrative level 4 counts. It is evident that both datasets produced using administrative level 3 data result in some over‐ and under‐estimations of population counts when assessed at administrative level 4. Unsurprisingly, the biggest differences are in Jakarta, where population totals are larger and vary more over shorter distances. Figure 6 does show, however, that generally lower levels of over and under‐estimation occur using the geotweet model.

**Figure 7:**
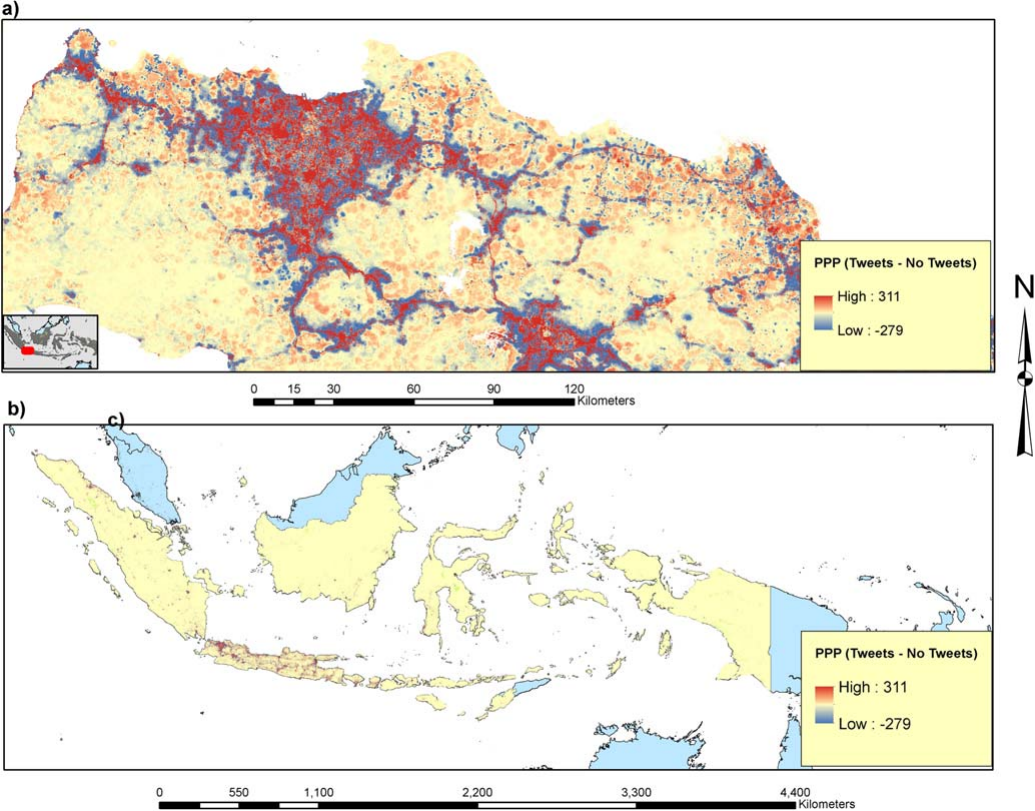
Difference map of persons per pixel (PPP) generated from subtracting the population map generated utilizing no geotweet data from the population map generated utilizing geotweet data: (a) Jakarta and surrounding areas; and (b) All of Indonesia

Figure 7 compares the final output population models (with and without geotweets) spatially, subtracting the geotweets model from the non‐tweets model to illustrate spatial patterns in differences. In addition to producing a more accurate model (Table 2, Figure 6), the figure highlights how the geotweets model concentrates populations into settlements more tightly, with less spread into more rural areas.

## 4 Discussion and Conclusions

Spatially‐disaggregated gridded population distribution datasets are becoming widely used, due principally to their flexibility in integration with other spatial datasets and summarization to any chosen level of aggregation. The accuracy with which this disaggregation can be achieved is related to the resolution and age of the input census data, but also to the quality, resolution and relevance of the spatial covariate layers used to statistically aid the disaggregation. The covariate layers typically used are often static in nature and their relationship to population densities can be unclear or interact in non‐linear ways. Despite these difficulties, high mapping accuracies can be achieved with suites of these static covariate layers (e.g. Stevens et al. 2015), but variance often still remains to be explained. Here we have shown that data from social media, representative of physical locations of people in space, represents a potential addition to these covariate options that can provide improvements in population mapping accuracies.

The results shown in Table 2 and Figure 4 demonstrate that the use of geotweet densities result in quantitative improvements in population mapping accuracies. Moreover, Figure 4 emphasizes that the geotweet density covariate was particularly important (third out of 30 covariates retained in the model) in contributing to the variance explained in the Random Forest models. With the number of Twitter users continuing to rise across the world, and the percentage of tweets that are geo‐located also rising as smartphones continue to proliferate, the results underline the potential of this data source in contributing to the improvement of population mapping and its dynamic update (Leetaru et al. 2013). Furthermore, other sources of social media data, some country specific like Baidu (China), Instagram, Shutterfly, and others also offer potential when the data is not only geospatially referenced but made available for research such as this.

While the results presented make a strong case for the integration of geotweet densities in improving population‐mapping accuracies, there are a number of caveats and drawbacks that should be addressed. First, Indonesia has one of the highest Twitter user rates in the world, making it an ideal setting for this test analysis (Leetaru et al. 2013). However, it remains unclear if similar results would be found elsewhere, particularly in areas such as sub‐Saharan Africa, where Twitter usage and smartphone penetration levels are much lower. Within Indonesia, there may also be geographical differences in mapping accuracies. Mapping improvements were only assessed by our analysis at the national level and sub‐national assessments may show areas of poorer mapping accuracies where Twitter usage levels are low. Furthermore, we see that population densities in final population maps are highly clustered around transportation networks, and potentially biased in this regard due to people using social media while in transport more frequently than when at home. Moreover, even though Twitter users share their exact location at the time of their tweets, the default spatial granularity of their tweets is set at “Neighborhood” level which is a geographic boundary defined by Twitter (https://dev.twitter.com/overview/terms/geo-developer-guidelines). As such, a high level of concentration of geotagged tweets with exact latitude and longitude was observed at aggregated points around city neighborhoods (Wu et al. 2015a, b). How this aggregation process by neighborhood affects fine scale population mapping still needs further assessment. Additionally, the impacts of demographic biases in Twitter account holders (e.g. they may represent younger segments of the population) remain unclear and warrant further exploration. However, the results overall showed that the geotweets made a positive contribution to mapping accuracies despite these caveats. Another factor that requires further exploration is the timing of the tweets. Here, tweets from all times of day were aggregated, representing a kind of “ambient” population distribution picture, which may not have been as representative of the residential population data from the census against which the outputs were used as a predictor. Further work should examine whether evening or nighttime‐only data provides a better representation of residential population, as has been shown previously for nighttime vs. daytime mobile phone call densities compared to census counts (Deville et al. 2014). Additionally, including sources of biasing due to age, income, time of day, smartphone availability, cost, and usage habits would make the results more informative and allow for exploration on how twitter data can be used in more diverse scenarios (Ramaswamy et al. 2009). Additionally, novel, open‐source social media applications are providing information that can be interpolated with population maps to generate better insights on the basic needs of individuals that exist within the gridded population counts (http://www.voicelots.com).

Future work will continue to examine the potential of geotweets in combination with other spatial datasets for improving population mapping. In particular, the integration of such data with mobile phone call and cell tower records offers potential for improving dynamic population mapping, and this will be explored for the 15+ low/middle income country call data record datasets being analyzed by the Flowminder Foundation (http://www.flowminder.org). Further, different methods of measuring and analyzing the geotweets will be undertaken, from varying time periods of capture, to differing spatial windows of aggregation. Integration with upcoming high resolution human settlement datasets will also be explored, including the Global Human Settlement Layer (http://ghslsys.jrc.ec.europa.eu/) and the Global Urban Footprint (http://dlr.de/eoc/en/desktopdefault.aspx/tabid-9628/16557_read-40454).

With the rise of smartphones and social media, the world population is transmitting more data on its presence and activities than ever before. Such data are often highly biased and incomplete, but nevertheless, this study has shown its potential in improving our understanding of human population distributions.
